# How to perform spectrum-based LC-HR-MS screening for more than 1,000 NPS with HighResNPS consensus fragment ions

**DOI:** 10.1371/journal.pone.0242224

**Published:** 2020-11-12

**Authors:** Anders Davidsen, Marie Mardal, Kristian Linnet, Petur Weihe Dalsgaard

**Affiliations:** Department of Forensic Medicine, Faculty of Health and Medical Sciences, Section of Forensic Chemistry, University of Copenhagen, Copenhagen, Denmark; Aarhus University, DENMARK

## Abstract

**Introduction:**

The ever-changing market of new psychoactive substances (NPS) poses challenges for laboratories worldwide. Analytical toxicologists are constantly working to keep high-resolution mass spectrometry (HR-MS) screening libraries updated for NPS. This study sought to use the online crowd-sourced HighResNPS database for spectrum comparison screening, thereby broadening its utility to all HR-MS instruments.

**Method:**

HighResNPS allows formation of a set of consensus fragment ions for a NPS and prioritises among multiple entries of collision-induced fragment ions. A subset of 42 NPS samples was analysed in data-independent acquisition (DIA) and data-dependent acquisition (DDA) modes on two different instruments. HighResNPS-computed spectra were generated with either Absolute (all fragment ions set to 100%) or Fractional (50% intensity reduction of former fragment ion) intensity. The acquired NPS data were analysed using the consensus library with computed ion intensities and evaluated with vendor-neutral screening software.

**Results:**

Overall, of the 42 samples, 100% were identified, with 88% identified as the top candidate. Three samples had the correct candidate proposed as the second highest ranking NPS. In all three of those samples, the top proposed candidate was a positional isomer or closely related compound. Absolute intensity assignment provided identical scoring between the top two proposed compounds in two samples with DIA. DDA had a slightly higher identification rate in the spectra comparison screening with fractional intensity assignment, but no major differences were observed.

**Conclusion:**

The fractional intensity assignment was slightly more advantageous than the absolute assignment. It was selective between proposed candidates, showed a high identification rate and had an overall higher fragmentation scoring. The candidates proposed by the HighResNPS library spectra comparison simplify the determination of NPS for researchers and toxicologists. The database provides free monthly updates of consensus spectra, thereby enabling laboratories to stay at the forefront of NPS screening by LC-HR-MS with spectra screening software.

## Introduction

The emergence of new psychoactive substances (NPS) on the illicit drug market is a challenge for forensic, toxicological, police and customs laboratories worldwide. The molecular diversity of the NPS and the ingenuity of the clandestine chemists complicate the task of detecting these novel compounds. The number of NPS on the drug market is increasing each year; meanwhile, previously known NPS enter and exit the drug scene continuously [1]. As a result, by the end of 2019, the United Nations Office on drugs and crime was monitoring more than 950 NPS [2]. In Europe alone, over 55 NPS were detected for the first time in 2018 [1].

Qualitative screening and detection of NPS can be performed on biological samples and law enforcement seizures. The screening procedures and techniques may vary between laboratories, but liquid chromatography (LC) coupled with high-resolution mass spectrometry (HR-MS) is a well-established methodology [3–5]. Screening for NPS by immunochemical assays is a possibility, though reduced specificity is a drawback [6]. For this reason, immunoassays do not provide component-resolved drug profiles for NPS, which may be important in clinical toxicology interpretations.

In forensic and clinical screening procedures, an analytical reference standard is essential for unambiguous identification; however, purchasing samples of every NPS is cost prohibitive, considering the volume and diversity of these compounds. Furthermore, reference standards are often not available for recently emerged NPS. This lack of good forensic and toxicological data tools means that newly emerging drugs may be overlooked. However, the use of external data sources can aid laboratories worldwide in the detection of emerging NPS by qualitative screening. These data sources may include data repositories such as the NPS data hub [7] or the RESPONSE database [8], or HR-MS databases such as Massbank [9], MzCloud (www.mzcloud.org/), or HighResNPS [10].

The current online databases vary in approach in terms of structure and organisation. HighResNPS is a free online crowd-sourced database intended for NPS screening with LC-HR-MS. The entries in HighResNPS are based on diagnostic fragment ions, as previously described [10, 11]. HR-MS/MS instruments tend to generate specific fragment ions after collision induced fragmentation of a common precursor ion [12, 13]; therefore, this database approach is favourable as it reduces the equipment and method differences. At present, HighResNPS has proven useful in HR-MS screening of law enforcement seizures [11] and environmental [14] and post-mortem [15] samples. Drug screening procedures may vary based on the software used, as a combination of diagnostic fragment ions and specific ion intensities is sometimes required for spectrum-based screening, in place of the diagnostic fragment ions alone.

The aim of the present study was to investigate the use of the HighResNPS database in spectra-based screening of NPS using HR-MS. The versatility of the spectra-screening database was examined using two commonly used HR-MS instruments and acquisition methods. A subset of 42 NPS was analysed using QTOF and Q-Orbitrap instruments in data-independent acquisition (DIA) and data-dependent acquisition (DDA) modes, respectively. The data were subsequently analysed by two spectrum comparison methods based on the HighResNPS consensus library. A secondary aim was to disseminate updates and improvements on the HighResNPS database beyond those described by Mardal et al. [10].

## Materials and methods

### Material and reagents

25IP-NBOMe, 3,4-dimethoxy-alpha-PHP, 4Cl-PVP, 4F-PHP, 4-MeO-PVP, alpha-PHP, 4F-NPP, DOF, DOIP, DOPR, MDPHP, NEB-indene-analogue (bk-IBP), N-Me-bk-MMDA-2, `3F-Phenetrazine, 4-AcO-MET, 4-MeO-BF, 5F-APP-PICA (PX1), 5F-PCN, 5-MAPDB, 5-MBPB, 5-MeO-DiBF, 5-PPDi, AB-CHFUPYCA, bk-2C-B, DB-MDBP, Isophenmetrazine, N-ethylphenmetrazol, CRL-40941, tBuONE, 25C-NBF, 4-Cl-PPP, PDM-35, HDMP-28, 4-AcO-DET, 3,6-DMPM, 3-MeO-PCMo, 4F-PV8, 4F-PV9, 4-CEC, 4-CIC, Ro5-4864, 5-MAPDI and 4-AcO-DMT were generously provided by the Slovenian National Forensic Laboratory and originated from the European project: “RESPONSE to challenges in forensic drug analysis”. The external calibration solution for positive ionisation was purchased from Thermo Fisher Scientific (Massachusetts, USA). The solvents used were of LC-MS grade or higher.

### Samples

The samples were test purchases of research chemicals or were collected from universities and had previously been identified by GC-MS, HPLC-TOF and/or NMR. Most samples were identified as pure, whereas the remaining had up to 25% contaminants. Datasheet and documentation for each compound are available on the RESPONSE website. All compounds were dissolved in MeOH upon arrival at the laboratory (1 mg powder/mL). The samples were diluted by a factor of 1000, resulting in an injection concentration of approximately 0.75–1.00 mg/L.

### Instrumentation

The analyses were conducted on two different instruments: a LC-quadrupole time-of-flight (QTOF) instrument (Waters, Manchester, UK) and a LC-Q-Orbitrap instrument (Thermo Fisher Scientific, Massachusetts, USA). The two instruments were operated with two different acquisition methods: the QTOF with data-independent acquisition (DIA) and the Q-Orbitrap with data-dependent acquisition (DDA). The analytical equipment and parameters used are presented in Table 1. All samples were analysed separately.

**Table 1:** Overview of the analytical equipment and parameters used for data-independent acquisition and data-dependent acquisition on QTOF and Q-Orbitrap instruments, respectively.

Parameters	Data-independent acquisition (QTOF)	Data-dependent acquisition (Q-Orbitrap)
Liquid chromatography	ACQUITY UPLC I-class (Waters)	Dionex Ultimate 3000 (Thermo Fisher)
Mobile phases	A: Aqueous 5 mM ammonium formate with 0.1% *v*/*v* formic acid	A: Aqueous 2 mM ammonium formate with 0.1% *v*/*v* formic acid
	B: Acetonitrile with 0.1% *v*/*v* formic acid	B: Acetonitrile with 0.1% *v*/*v* formic acid
Gradient	13% to 50% B at 10 min, 50% to 95% at 10.75 min and a flow rate at 0.4mL/min	13% to 50% B at 10 min, 50% to 95% at 10.75 min and a flow rate at 0.4mL/min
Column	Acquity HSS C18 1.8μm, 2.1 × 150 mm (Waters) at 50°C	Acquity HSS C18 1.8μm, 2.1 × 150 mm (Waters) at 50°C
Injection volume	3 μL	5 μL
Trade name of HR-MS	Xevo G2-S QTOF (Waters)	Q-Exactive (Thermo Fisher)
Acquisition mode and parameters	Data-independent acquisition: MS^E^, scan range: 50–950 *m/z*. Nebulisation gas 1000 L/h with a temperature of 400°C. Cone gas flow 20 L/h. Source temperature 150°C. Capillary voltage 800 V; cone voltage 25 V.	Data-dependent acquisition: resolution of 35,000, AGC at 3e6 ions, IT of 50ms, scan range: 100–900 *m/z*, ddMS^2^: Resolution of 17,500, AGC 1e5, maximum IT of 50 ms, isolation window of 1 *m/z*. Dynamic exclusion: 5 s.
Ionisation technique	Positive electrospray ionisation	Positive heated electrospray ionisation
Collision gas	Argon	Nitrogen
Collision energy	Low collision energy (4 eV) and high collision energy with ramp: 10eV to 40eV	Normalised collision energy: 20, 35, 50
Calibration	External mass calibration with 5 mM sodium formate solution in propanol:water (90:10, *v/v*). Internal lock mass was conducted with leucine enkephalin	External calibration solution

*IT: Injection time, AGC: Automatic gain control.

### HighResNPS consensus fragment ions

The basic structure and architecture of HighResNPS have previously been described by Mardal et al. [10], and the functionality of the online database was described by von Cüpper et al. [11]. HighResNPS consensus fragment ions are a new feature of HighResNPS that was recently implemented and not previously reported.

The grouping of multiple NPS entries for one drug is achieved by the first 14 characters in the structure-specific identifier: the InChIKey (https://iupac.org/who-we-are/divisions/division-details/inchi/). These 14 characters define the molecular structure and disregard the stereochemistry, as this information is encoded in the last part of the InChIKey. This allows the grouping of fragment ions for multiple entries per compound, based on a frequency-based prioritisation mechanism. All entries have reported up to three fragment ions, ranked from their highest observed intensity to the third highest observed intensity. Prioritisation considers both the ranking and frequency of the entries in the database and allows up to a maximum of six consensus fragment ions.

The transformation from eight entries of the synthetic cannabinoid receptor agonist AB-FUBINACA from HighResNPS.com to the ranked consensus fragment ions is shown in Table 2.

**Table 2:** Conversion of fragment ion entries on the website into HighResNPS consensus fragment ions for an NPS. The prioritisation is based on the entry order of the fragment ions. The HighResNPS consensus fragment ions are shown at the bottom of the table.

Entry	InChIKey			Precursor [M+H]	Fragment 1 (3 points)	Fragment 2 (2 points)	Fragment 3 (1 points)
User 1 (Bruker)	AKOOIMKXADOPDA-KRWDZBQOSA-N			369.1721	253.0772	109.0448	324.1506
User 2 (Waters)	AKOOIMKXADOPDA-KRWDZBQOSA-N			369.1721	109.0448	253.0772	324.1506
User 3 (Thermo)	AKOOIMKXADOPDA-KRWDZBQOSA-N			369.1721	253.0772	271.0877	109.0448
User 4 (Thermo)	AKOOIMKXADOPDA-KRWDZBQOSA-N			369.1721	253.0772	109.0448	324.1506
User 5 (Agilent)	AKOOIMKXADOPDA-KRWDZBQOSA-N			369.1721	324.1506	352.1456	253.0772
User 6 (Agilent)	AKOOIMKXADOPDA-KRWDZBQOSA-N			369.1721	253.0772	324.1506	109.0448
User 7 (Waters)	AKOOIMKXADOPDA-UHFFFAOYSA-N			369.1721	253.0772	324.1506	109.0448
User 8 (Bruker)	AKOOIMKXADOPDA-UHFFFAOYSA-N			369.1721	324.1506	352.1456	391.1540
**HighResNPS consensus fragment ions**							
**Name**	**Precursor [M+H]**	**F1**	**F2**	**F3**	**F4**	**F5**	**F6**
AKOOIMKXADOPDA	369.1721	253.0772	324.1506	109.0448	352.1456	271.0877	391.1540
Points		18	13	10	6	2	1

### Identification of NPS

The HighResNPS consensus fragment ions are transformed to spectra by assigning computed ion intensities. Two different methods of ion intensity assignment were asserted:

Absolute intensity: all fragment ions are assigned an ion intensity of 100% and

Fractional intensity: each ion intensity was reduced by 50% of the former fragment ion.

All entries for a NPS get a fragment ion intensity assigned, regardless of the number of entries.

Analytical data for the NPS from both instruments were added to HighResNPS. Subsequently, the acquired data were analysed using the consensus library from the database. Before making online entries of the analytical data, a minimum of one entry from another laboratory existed in the HighResNPS database for each of the 42 NPS.

Data files from the QTOF were handled by UNIFI 1.9.2 (Waters, Manchester, UK), while data generation and handling from the Q-Exactive was conducted using Xcalibur 4.1 (Thermo Fisher Scientific, Massachusetts, USA). All acquired data were evaluated on the instrument-neutral software platform Progenesis QI (Nonlinear dynamics, Massachusetts, USA). The unprocessed data files were imported directly into Progenesis QI by selecting the appropriate file type (Raw: Thermo or UEP: Waters). To use Progenesis QI (version 2.4.6911), the HighResNPS library was downloaded from HighResNPS.com as an Excel file containing consensus library fragment ions (HighResNPS_CONSENSUS_April_2020.xlsx). Python (version 3.6.8) and ChemAxon (https://chemaxon.com/ JChem for Office 18.20.0.353, 2018) were then used to convert the Excel consensus library to a Structure Data File (sdf) from NPS SMILES, an absolute ion intensity file (msp) and a fractional ion intensity file (msp). These sdf and msp files were then used for Progenesis QI comparison of spectra linked by the InChIKey. The sdf file was used to detect the precursor ion with a 10 ppm mass error and fragment ions with a 20 ppm mass error. Progenesis QI compound scoring (from 0 and increasing to 100) was generated by fragmentation similarity between the spectra from the computed intensities assigned to the HighResNPS consensus fragment ions and the acquired spectrum. The fragmentation scoring in Progenesis QI is based on the cosine similarity method, which calculated the dot product of two vectors i.e. the experimentally obtained spectrum and the database spectrum. Figures were created in Inkscape and Python.

## Results

### Spectrum comparison

**Fig 1:**
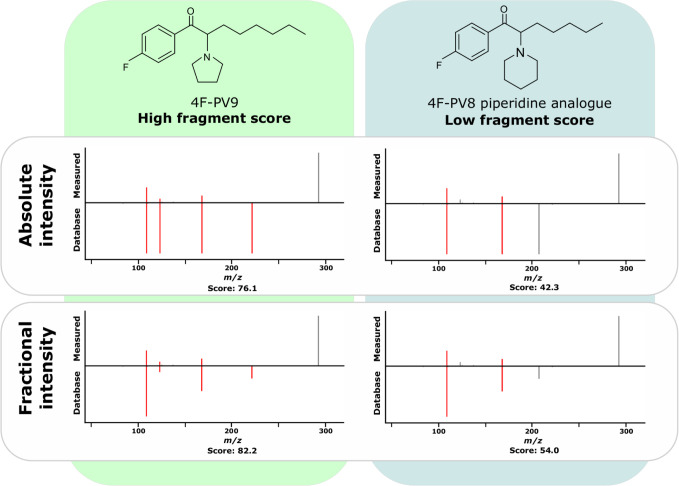
**Illustration of spectral comparison using the software Progenesis QI for the isomers 4F-PV9 (left) and 4F-PV8 piperidine analogue (right) obtained in data-independent acquisition for absolute intensity (top) and fractional intensity assignment (bottom).** Positively identified fragment ions are marked in red. The assigned fragment scores are shown below the spectra.

The acquired spectra were matched against the HighResNPS spectral databases. An example of a spectra comparison is shown for 4F-PV9 and 4F-PV8 piperidine analogues in Fig 1. There, the absolute intensity and fractional intensity spectra are compared and scored against the acquired QTOF spectrum.

The full screening result table is available in the supporting information (S1 Table). In total, 88% (n = 37) of the investigated NPS acquired on the two instruments with two types of spectra were correctly proposed as the top candidate. Of the 42 samples, three samples had the correct candidate proposed as the second highest ranking NPS. In the three cases the top candidate proposed was a positional isomer or closely related compound. One sample, 5F-PCN, was wrongly assigned as the azaindol-derived isomer 5F-MN-18 in DIA with both ion intensity assignment methods. The two proposed compounds share the three fragment ions with m/z 233.1085, 213.1022 and 145.0396. Similarly, DOPR was wrongly assigned as the isopropyl analogue DOIP by the fractional method on both instruments as the two compounds share fragment ion m/z 221.1536, 206.1301 and 193.1223, leaving one compound specific m/z 164.0832 for DOIP. Lastly, 4-methoxybutyrfentanyl was wrongly assigned as the positional isomer 2-methoxybutyrfentanyl by the absolute intensity method on both instruments due to the similar fragmentation pattern with two overlapping fragment ion m/z. Samples containing 4-chlorodiazepam and 25IP-NBOMe were each assigned as two possible drug candidates with identical scores by the absolute intensity method. Based on the monoisotopic mass of the investigated NPS the number of possible isomers on HighResNPS ranged between 1 and 22 once only searching by m/z. The spectrum-based screening greatly reduced the number of potential NPS candidates.

**Fig 2:**
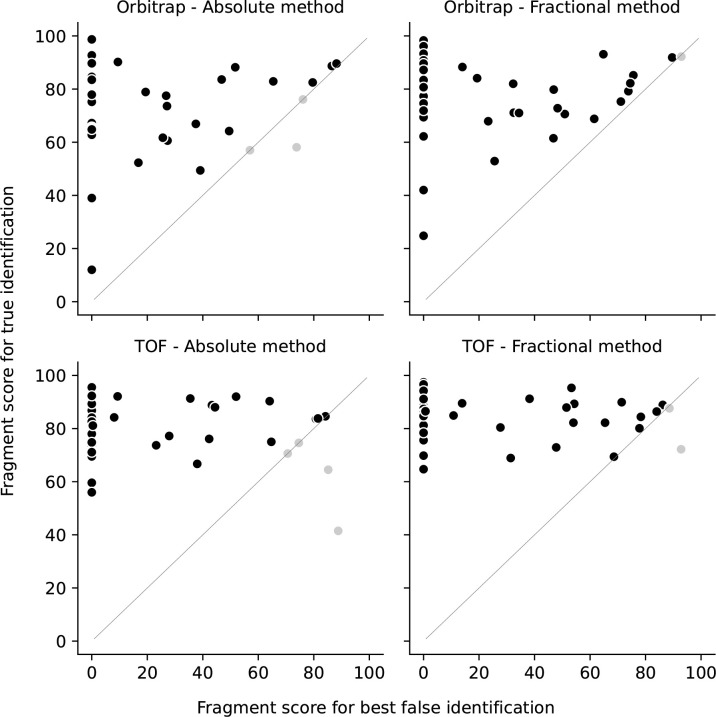
Fragment score for the best false identification plotted against the fragment score for the true identifications for the two acquisition methods and ion intensity generation methods. Black markers show correctly identified NPS, whereas grey markers show incorrectly identified NPS. The grey diagonal lines indicate whether the sample is correctly or falsely proposed (n = 42).

The mean scoring of the correctly identified compounds in DDA were 74.6 ± 16.7 and 78.4 ± 14.5 (mean ± SD) for the absolute intensity and the fractional intensity methods, respectively. The mean scoring for data obtained with DIA for absolute and fractional intensity assignment were 80.1 ± 11.3 and 84.2 ± 8.18, respectively. The fragment score for best false identification is plotted against the true identification fragment score in Fig 2. If only one NPS was proposed by Progenesis QI, the value of the best false identification was set to 0. Generally, the fragment score was higher with DIA data than with DDA data, with fractional intensity assignment providing the highest score in both acquisition modes. The identification rates for data obtained with DDA were 92.9% and 97.6% for absolute and fractional intensity assignments, respectively. The identification rates for data obtained with DIA were 90.5% and 95.2% for absolute and fractional intensity assignments, respectively. The largest percentage difference between the correct NPS and the best false proposed candidate was most frequently observed with the absolute intensity assignment method. This was observed in both acquisition modes.

### HighResNPS dataset

**Fig 3:**
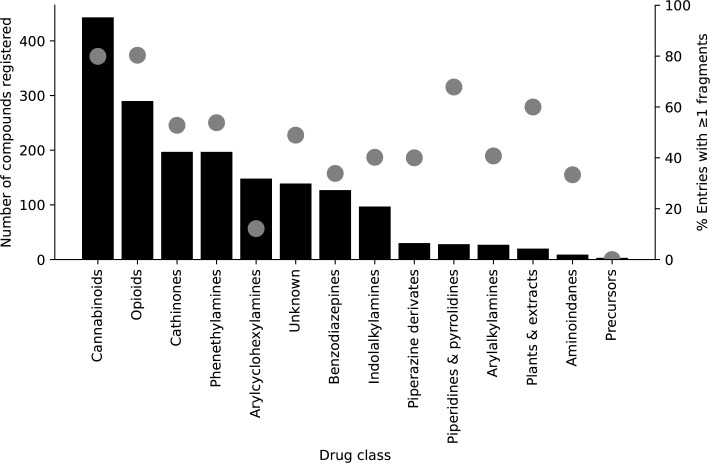
Illustration of the number of compounds registered for different new psychoactive substance classes as a bar chart (left y-axis) and the percentage of each compound class with one or more fragment ions registered in HighResNPS as grey symbols (right y-axis).

The HighResNPS dataset used in the present study was from April 2020 and contained data from 28 laboratories located in 16 different countries. Based on 2795 entries, the current total number of compounds in the database is 1755. A visual representation of the NPS class entries and the percentages of one or more fragment ions registered within the drug class is shown in Fig 3. The drug class is based on the European Monitoring Centre for Drugs and Drug Addiction (EMCDDA) categorisation.

The synthetic cannabinoid receptor agonists and opioids are the two most reported classes in the database in terms of both the number of entries and the percentage having more than one fragment ion registered. Across all drug classes and entries in the database, more than 58% of the entries had one or more fragment ions registered. Of the 1022 compounds having fragment ions registered, more than 45% had fragmentation data from more than one source.

## Discussion

As the number of online compound databases have increased the last years, the performance of HR-MS instrumentation and proposals for quality control of the databases have likewise come into focus. The improvement of instrument performance and better database standardisation have facilitated the transferability of detected diagnostic fragment ions among databases, thereby promoting overall the usability and robustness of databases [12, 13, 16].

Historically, immunoassays have been useful in detection of drugs of abuse and new methods are continuously being developed [17]. Several emerging NPS enter the market yearly, and may be pose challenges for identification rate and high cut-off concentrations with immunoassays [18, 19]. The rapidity at which NPS enter and exit the drug market makes HR-MS libraries increasingly favourable, as they are rapidly updated with newly detected NPS [6, 20]. Immunoassays can be easier to fit into a clinical workflow operating 24/7, especially as an LC-HR-MS NPS screening method may require personnel with greater skills. However, having access to comprehensive high-quality HR-MS screening yields more clinically useful results due to the component-resolved drug profile.

In the present study, spectra based on computed ion intensities of HighResNPS consensus fragment ions were evaluated and discussed based on the software-specific scoring number from Progenesis QI. The mean scoring differences between data obtained with DIA and DDA may be attributed to several factors as instrumentation differences, collision energy, Progenesis QI scoring algorithm bias and/or mass accuracy variation. In a clinical setting, with screening of authentic samples, what matters is not an arbitrary number, but the screening library’s ability to correctly identify and easily distinguish true from false positive identifications. In the present work, the fragment scoring is based on analysis of neat samples and is therefore not representative of values obtained in authentic sample screenings with complex matrices. Nevertheless, the high identification rate in this study emphasises that different instrumentation and analytical methods can be used successfully with the HighResNPS database, as previously described [10, 11, 14, 15]. In addition, this is now also verified for spectrum-based screening software. An increase in fragment score assignment may be achieved by including precursor ions in the computed spectra screening, as the precursor ion often is present in the product ion spectra. However, including precursor ions may also lead to more false positive identifications, as fragment ions in the background or matrix will be identified in both the precursor and product ion spectra. Furthermore, all isomers will have the precursor ion as a positive product ion, which will further increase the number of false positive identifications. The strength of the utilisation of HighResNPS in spectral comparison screening is shown by its differentiation between the three structurally diverse isomers 3,6-DMPM, PDM-35, and 5-MAPD. These three compounds were correctly identified with the proposed candidate based on the reported fragmentation, despite all having identical m/z values.

The fragment score difference between the top two proposed candidates was highest for most samples with absolute intensity assignment. This indicates that this method presumably also distinguishes best between the proposed candidates. The work conducted by Gundersen et al. also suggested that absolute intensity assignment is suitable in retrospective analysis of post-mortem blood samples [15], thereby showing utility of the database when analysing complex biological samples. However, in the present study, the absolute intensity assignment method resulted in identical scoring for the two top proposed compounds for several of the NPS investigated, thereby not always differentiating between the proposed compounds. The similar results obtained between two very different relative ion intensity methods suggest that the accuracy of diagnostic fragment ions are prioritised over ion intensity in Progenesis QI spectral match comparison. Nevertheless, the spectrum comparison always will be manually inspected, so the fractional intensity assignment appears to have a slight advantage over absolute intensities and can thus be recommended for computed spectrum comparisons.

Spectrum-based screening algorithms vary between screening software with different weightings of ion intensity. For this reason, we strongly recommend that users test the software sensitivity for ion intensity to see which computed spectra work better on their screening software. If other computed ion intensities are desired by users of the database, this is possible by modifying the downloaded msp file. Computed ion intensities are required in some vendor software to allow importation of a screening library; therefore, the assignment of computed ion intensities will broaden the target audience of the HighResNPS database and possibly increase the detection rate in screening protocols for NPS.

The matrix varies in NPS screening depending on whether the origin of the sample is a seizure or a biological material. This means that the samples may have varying degrees of complexity, which may complicate identification of the compounds. The identification confidence and/or reduce potentially false positive identifications can be increased by implementing a parameter like the retention time [21]. In those cases, HighResNPS may be utilised in combination with predicted retention times, as described by Diamanti et al. [14].

In general, the HighResNPS database is a powerful tool for NPS screening as the database is continuously updated with entries by the participants. Nonetheless, caution should be taken as screening with the consensus library will not allow differentiation between positional and closely related isomers. The detected fragment ions may be similar and may be differentiated only by chromatographic separation or ion intensities. Therefore, the consensus library ought to be used as a suspect screening library only, and positive hits should always be confirmed with complementary techniques and/or analytical standards.

The entry frequency of synthetic cannabinoid receptor agonists and opioids in HighResNPS appears to reflect the trends in the drug market, as this overrepresentation is in accordance with the last few years of EMCDDA drug reports [1, 22]. The strength of the HighResNPS database relies on multiple users adding analytical data, including data on compounds which have previously been registered. The availability of consensus fragment ions now makes possible the full harnessing of the power of having multiple entries from users with different analytical conditions. When multiple laboratories with instrument and/or method differences contribute with fragment ions for the same compound, the entered fragment ions are ranked based on frequency for the given NPS. As a result, the credibility of the top-prioritised fragment ions increases with the number of participating laboratories, and the noise of irrelevant or wrongly assigned fragment ions decreases. This down-prioritisation of wrongly entered diagnostic fragment ions in the ranking makes the database more error resilient. For application uses, the consensus library is updated monthly and added to the homepage. More importantly, the new updates are available for download for different types of HR-MS screening software.

## Conclusion

In this paper we have demonstrated how the HighResNPS consensus library can be of use in spectrum-based screening by computing ion intensities. Two different acquisition modes were used to acquire data and two different methods were tested to assign ion intensity on the data set. Of the 42 NPS samples, 100% were identified within the top two candidates and 88% were the top candidate. The wrongly proposed top candidate in the three samples were all positional or closely related isomers. The fractional ion intensity assignment method proved most useful, due to a higher identification rate and usability in visual spectrum comparison. The updated HighResNPS database affords the opportunity to download and use the consensus library in screening methods for more instruments and screening software, while utilising collision induced fragmentation prioritisation to increase identification confidence. The crowd-sourced database provides a self-verification, as multiple entries on the same compound will down-prioritise wrongly indicated diagnostic fragment ions. This study demonstrates how to perform spectrum-based LC-HR-MS screening for more than 1,000 NPS with HighResNPS consensus fragment ions.

## Supporting information

S1 Table The raw data has been uploaded to Mass spectrometry Interactive Virtual Environment (MassIVE) with doi: 10.25345/C5BV20. (XLSX)
